# The longitudinal dynamics evolution of optical skyrmions via meta-optics

**DOI:** 10.1515/nanoph-2025-0436

**Published:** 2025-12-01

**Authors:** Tiantian He, Chang Liu, Wenxuan Tang, Dan Li, Ping Yan, Qiang Liu, Qirong Xiao

**Affiliations:** Department of Precision Instrument, 12442 Tsinghua University, No. 1 Qinghua Garden, Chengfu Road, Haidian District, Beijing 100084, P.R. China; State Key Laboratory of Precision Space-time Information Sensing Technology, No. 1 Qinghua Garden, Chengfu Road, Haidian District, Beijing 100084, P.R. China

**Keywords:** Skyrmions, metasurfaces, structured light, topology

## Abstract

Skyrmions, as topologically structured light fields, have attracted considerable attention due to their unique topological properties and potential applications such as optical communication and advanced sensing technologies. However, their longitudinal evolution, as a dimension ripe for exploitation, typically remains uncontrolled and non-deterministic, hindering its in-depth exploration and application scenarios. Here, this paper presents a novel method using dielectric metasurfaces for precisely modeling the longitudinal dynamics evolution of skyrmions. We introduce a new mechanism that allows for the accurate period modulation of skyrmions stokes properties along the propagation direction by controlling the differences in numerical apertures of a zero-order right-circularly polarized beam and a first-order left-circularly polarized beam. Crucially, the evolution period can be arbitrarily designed, and the propagation distance can be expanded by increasing the waist radius of input beams. To validate this approach, we showcase this paradigm through displacement sensing applications, where single-snapshot polarization measurements directly infer absolute position within a compact metasurface-integrated platform, offering a compact and simple alternative to conventional scanning-based approaches for displacement sensing. Our approach advances the understanding of dynamically controlled topological light fields and enables compact devices for precision metrology and optical information technologies.

## 1 Introduction

The skyrmion, a topological soliton first proposed by Tony Skyrme in 1962 [1], has gained significant attention in condensed matter physics [2] and magnetic field [2], [3], [4] due to its topological protection. This interest has further extended to optics, where optical skyrmions [5], [6], [7], [8], constructed using electromagnetic fields, show great potential for applications in optical communication, sensing technologies [9], and super-resolution positioning [10]. In particular, the Stokes skyrmions [5], defined by the polarization state of light, represent light fields with nontrivial topological structures and offer a promising approach for manipulating and transferring topological information over long distances in optical systems.

Consequently, significant research efforts have been devoted to generating optical skyrmions [5], characterizing their topological structures [11], [12], and understanding their propagation-induced transformations [13], primarily explored at specific planes [12], [14] or fixed propagation distances [15]; recent advances have further extended to three-dimensional topological volumes [11]. Experimental and theoretical advances have yielded deep insights into their intricate spin configurations [12], [13], [16], quantized topological charges, and resilience [17] within confined near-field regions [18], [19] or at designated observation planes. The longitudinal evolution of such skyrmions has been demonstrated in systems based on Laguerre–Gaussian (LG) beams, where the dynamics are confined by a non-linear Gouy phase [20]. In contrast quasi-nondiffracting beams can support a linear phase evolution with distance, enabling extended and uniform dynamics [21], [22].

Although significant progress has been made in the study of static or quasi-static optical skyrmions, their longitudinal evolution, a key aspect of their spatiotemporal dynamics, remains largely unexplored [12], [14], which refers to how the properties of skyrmions [23], such as their polarization and topological characteristics, evolve along the propagation direction. In most existing systems, this evolution is non-deterministic, which limits the practical use of skyrmions in applications such as displacement sensing and high-precision metrology. Many current generation methods rely on homogeneous numerical apertures (NAs), which suppress longitudinal dynamics due to wavevector degeneracy. Some recent approaches, like those using digital micromirror devices [12], offer control of the propagation dynamics of light field $E\left(x,y,z\right)$ , but they depend on bulky free-space optics and multiple polarization elements, hindering integration and scalability. Skyrmions are typically generated through complex setups such as spatial light modulators [24], making real-time control and position feedback difficult. While there has been progress in studying skyrmions in confined systems, controlling their behavior over extended distances, is still in its early stages. So far, deterministic control of their longitudinal evolution within a compact, integrated platform has yet to be realized, highlighting a significant area for further exploration.

Metasurfaces [25], [26], [27], [28], [29], composed of arrayed subwavelength nanostructures that locally tailor the phase [30], amplitude, and polarization of light [31], [32], have emerged as powerful tools [28] for manipulating structured beams [33], [34] and generating optical vortices [35], [36]. Their planar geometry, ultrathin profile, and design flexibility make them attractive for integration into compact photonic systems. However, their use in dynamic, high-precision control of skyrmion evolution along the propagation axis remains limited. Current metasurface-based methods typically generate static skyrmions, lacking tunability and precise longitudinal control.

Recent studies have demonstrated that metasurfaces can directly generate optical skyrmions [37], [38], [39], offering compact and efficient means to realize topological light fields without relying on bulky free-space components. However, they are mostly limited to static skyrmion patterns, without addressing how these structures dynamically evolve along the propagation direction. This lack of longitudinal control limits their functionality in applications that require tunable beam dynamics, such as sensing, trapping, or information encoding. To fully realize the potential of metasurface-based topological photonics, it is therefore crucial to develop strategies that enable both the generation and precise, reconfigurable modulation of skyrmions along the axial direction within an integrated platform.

In this paper, we propose a novel approach for modeling and controlling the longitudinal evolution of skyrmions using dielectric metasurfaces. A monolithic dielectric metasurface generates interfering left-circularly polarized (LCP) and right-circularly polarized (RCP) Bessel beams of different orders, creating a skyrmion. The Stokes fields of this skyrmion can be precisely and periodically controlled, with the evolution period adjustable by manipulating the NAs of the LCP and RCP beams. By adjusting the longitudinal wavevector mismatch, we can modulate the periodicity of the light’s topological properties, offering a powerful tool for high-precision light control. This work also demonstrates the effectiveness of the proposed method through simulation-based displacement sensing. Following metasurface design and subsequent calibration procedures, we can realize absolute position sensing with a single snapshot of polarization data, showing notable improvements in integration and portability compared to conventional displacement sensing techniques. Using the Bessel beam foundation and longitudinal skyrmion evolution, our method achieves nanometer-scale precision and an extended operational range. This method not only advances the understanding of dynamically controlled topological light fields but also provides a compact platform for optical metrology and optical information technologies such as structured-light communication and polarization-based data encoding.

## 2 Methods and results

### 2.1 Optical skyrmions: fundamental concepts and dynamics evolution

**Figure 1:**
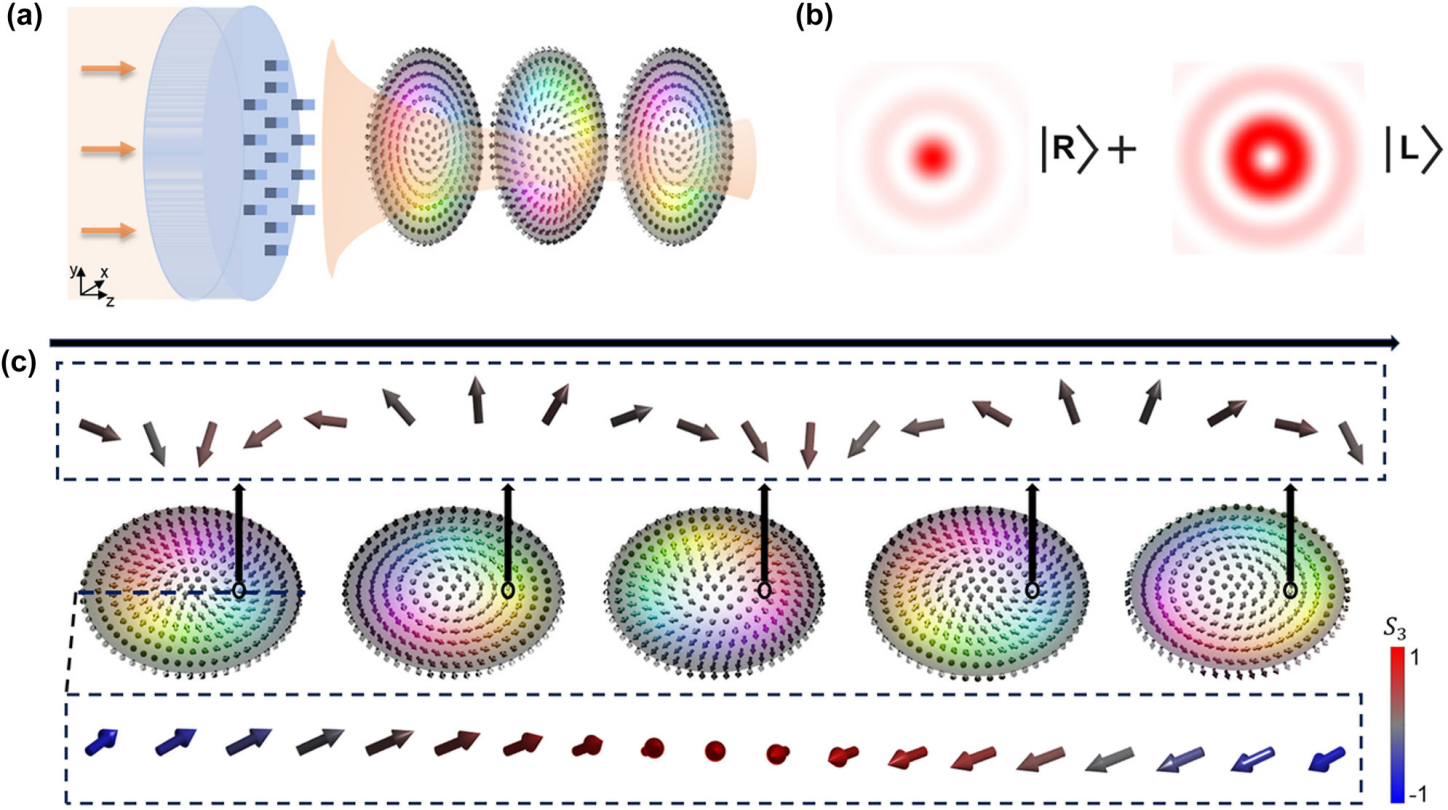
The principles of generating optical skyrmions via metasurface. (a) Schematic illustration of the generation and propagation of optical skyrmions via a metasurface. Linearly polarized light passes through a spin–orbit coupling metasurface, generating structured beams with evolving polarization textures along the propagation direction. The polarization texture undergoes periodic transformations. (b) Principle of skyrmion generation based on the inseparable superposition of a zeroth-order Bessel beam with right-handed circular polarization and a first-order Bessel beam with left-handed circular polarization. (c) Polarization evolution of the optical skyrmion along the *z*-axis. The central row shows hue-colored polarization distributions at five selected axial positions, while the top row illustrates the corresponding periodic rotation of polarization vectors along a circular cone trajectory. The bottom row presents polarization distributions extracted along a line at selected cross-section. The color of each vector indicates the value of the Stokes parameter *S*_3_, as shown by the color bar, ranging from left-handed (blue) to right-handed (red) circular polarization.

The propagation of the synthesized optical field is characterized by a continuous and periodic evolution of its internal polarization structure. This dynamic behavior is qualitatively illustrated in Figure 1. The central panel displays the transverse cross-sections of the beam at several incremental propagation distances along the *z*-axis. Each plane exhibits a topologically non-trivial polarization distribution, known as a Stokes skyrmion, where the color and small arrows represent the local orientation of the transverse Stokes vector $\left(S_{1},S_{2}\right)$ . As the field propagates, this entire skyrmionic texture is observed to undergo a rigid, periodic transformation.

This longitudinal evolution is explicitly visualized in the top panel, which tracks the full three-dimensional Stokes vector, $S=\left(S_{1},S_{2},S_{3}\right)$ , at a single fixed point in the transverse plane. The vector undergoes a clear periodic precession, completing two full cycles over the distance shown. Furthermore, the internal structure within any single plane exhibits a high degree of order. Together, these visualizations illustrate the periodic transformation of the field’s topological polarization state.

### 2.2 The principles of longitudinal evolution of skyrmions

The theoretical foundation of the longitudinal evolution is based on the coaxial superposition of two Bessel-Gauss (BG) beams [40], constructed within an orthogonal right-circular ( $\left|R\right\rangle$ ) and left-circular ( $\left|L\right\rangle$ ) polarization basis. The choice of Bessel–Gauss (BG) beams is fundamentally guided by their Gouy phase characteristics. Unlike the nonlinear dependence that dictates evolution in Laguerre–Gaussian (LG) systems [20], the effective Gouy phase in quasi-non-diffracting BG beams can be designed to evolve linearly with propagation [21]. This linear evolution is pivotal, as it enables the uniform and continuous polarization transformation we demonstrate across extended distances. The total optical field, represented by the Jones vector $\left|\Psi \right\rangle$ , is mathematically expressed as:

$$\begin{array}{c} \left|\Psi \right\rangle ={BG}_{l_{R}}\left(r,\phi ,z,k_{z}^{R}\right)\left|R\right\rangle +{BG}_{l_{L}}\left(r,\phi ,z,k_{z}^{L}\right)\left|L\right\rangle \end{array}$$

Here ${BG}_{l_{R}}$ and ${BG}_{l_{L}}$ are the complex amplitudes of the right- and left-circularly polarized BG beams, respectively. Each beam is characterized by its topological charge, denoted by lR and lL. The general form for the complex amplitude of a BG beam with topological charge *l* is given by:

$${BG}_{l}\left(r,\phi ,z,k_{z}\right)=e^{-r^{2}/w_{0}^{2}}J_{l}\left(k_{r}r\right)e^{il\phi }e^{ik_{z}z}$$

where, *J*_*l*_ is the *l*th-order Bessel function of the first kind, which describes the beam’s radial profile, while the term *e* ^*ilϕ*^ represents the helical phase front corresponding to the topological charge *l*. The term $e^{-r^{2}/w_{0}^{2}}$ is a Gaussian envelope with beam waist *w*_0_, which ensures the beam has finite energy and is physically realizable. In our model, the term $e^{ik_{z}z}$ represents an effective, linearized axial phase. It is a valid approximation of the exact, non-linear Gouy phase of a Bessel–Gauss beam under the condition that the propagation distance *z* is much smaller than the Rayleigh range *L* of the input Gaussian envelope (*z* ≪ *L*), which is satisfied in our design. Within this regime, nonlinear contributions are negligible, resulting in a linear on-axis phase progression. This linearization is indispensable, as it establishes the stable, periodic beating governed by Δ*k*_*z*_ *z*, that drives the skyrmion evolution.

The propagation dynamics and local polarization structure of the synthesized beam are best understood by analyzing its Stokes parameters. After substituting the field components into the standard Stokes definitions, we obtain:

$$\begin{array}{c} s_{0}=J_{l_{R}}^{2}\left(rk_{r}^{R}\right)+J_{l_{L}}^{2}\left(rk_{r}^{L}\right)\\ s_{1}=2J_{l_{R}}\left(rk_{r}^{R}\right)J_{l_{L}}\left(rk_{r}^{L}\right)\cos\left(\Delta l\phi +\Delta k_{z}z\right)\\ s_{2}=-2J_{l_{R}}\left(rk_{r}^{R}\right)J_{l_{L}}\left(rk_{r}^{L}\right)\sin\left(\Delta l\phi +\Delta k_{z}\right)\\ s_{3}=J_{l_{R}}^{2}\left(rk_{r}^{R}\right)-J_{l_{L}}^{2}\left(rk_{r}^{L}\right) \end{array}$$

Note that the Gaussian amplitude factor $e^{-r^{2}/w_{0}^{2}}$ common to all field components has been omitted from the Stokes parameters for clarity. This simplification is valid because the skyrmionic texture is defined by the normalized Stokes vector, *S*_*i*_ = *s*_*i*_/*s*_0_ (*i* = 1, 2, 3), wherein this common factor cancels out exactly. Thus, the envelope only affects the total intensity $S_{0}\left(r\right)$ without altering the local polarization state.

These equations form the complete analytical basis of the field’s behavior. A detailed analysis of their structure reveals the dual nature of the encoded information. The total intensity *S*_0_ and the circular polarization component *S*_3_ are functions of the radial coordinate but are independent of the propagation distance *z*. This longitudinal invariance of *S*_3_ is particularly important, as it defines a stable ring of pure linear polarization at the radius where $J_{l_{R}}=J_{l_{L}}$ , which serves as a natural frame for our analysis. In contrast, the linear Stokes components *S*_1_ and *S*_2_ contain a phase term, Δ*lϕ* + Δ*k*_*z*_ *z*, that explicitly couples the transverse azimuthal coordinate *ϕ* with the longitudinal coordinate *z*. The term Δ*lϕ* (with Δ*l* = *l*_*L*_ − *l*_*R*_ = 1) governs the transverse vector field, giving rise to the skyrmionic texture. The term Δ*k*_*z*_ *z* governs the longitudinal evolution, driving the periodic precession of this entire texture as it propagates.

To realize a controlled longitudinal periodic evolution of the Stokes textures, we relate the axial beating period Λ directly to the NA of the two superimposed Bessel beams. For a medium of refractive index *n* and wavelength *λ*, the axial wavevector of a beam is:

$$k_{z,i}=\frac{2\pi }{\lambda }\sqrt{1-{NA}_{i}^{2}}$$

The difference Δ*k*_*z*_ = *k*_*z*1_ − *k*_*z*2_ governs the axial beating of the Stokes vector, giving the period

$$\Lambda =\frac{2\pi }{\Delta k_{z}}$$

Accordingly, one can design the period by selecting appropriate values of NA_1_ and NA_2_. Conversely, for a target period Λ, the required NA_2_ (given NA_1_) can be obtained as

$${NA}_{2}=\sqrt{1-{\left(\sqrt{1-{NA}_{1}^{2}}-\frac{\lambda }{\Lambda }\right)}^{2}}$$

In practice, the visible number of periods is further limited by the non-diffracting length of the two Bessel beams, the effective number of observable periods is estimated as $N_{per}\approx \frac{\min\left(z_{nd,1},z_{nd,2}\right)}{\Lambda }$ .

**Figure 2:**
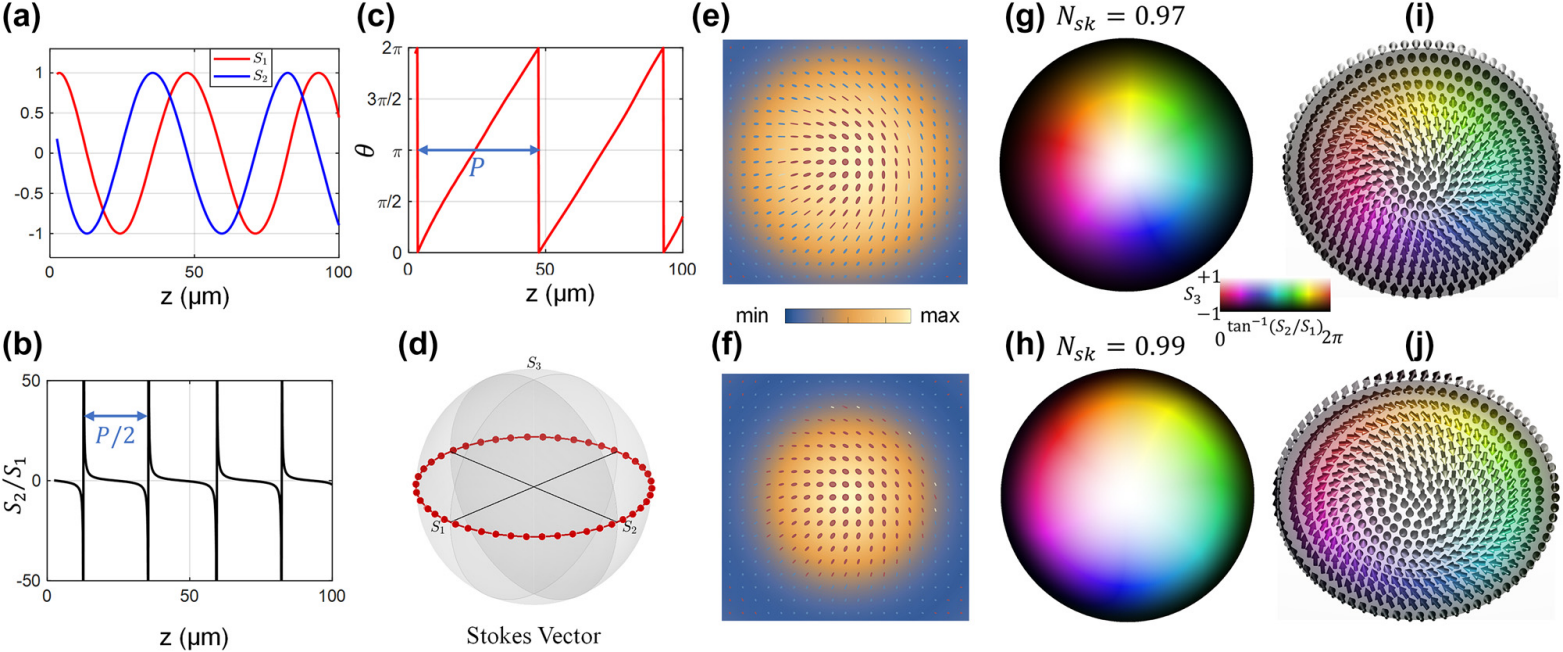
Theoretical and numerical analysis of the periodic longitudinal evolution of the Stokes skyrmion. (a–d) Quantitative analysis of the periodic precession at the *S*_3_ = 0 radial ring. (a) Normalized Stokes parameters *S*_1_ and *S*_2_ versus propagation distance *z*. (b) The corresponding ratio *S*_2_/*S*_1_ versus *z*, which highlights the half-period (*P*/2) symmetry, with singularities occurring where *S*_1_ is close to 0. (c) The azimuth angle of the Stokes vector versus *z*, showing a linear increase over each period *P*. (d) Trajectory of the Stokes vector on the Poincaré sphere. (e–j) Visual confirmation of periodicity by comparing the field at two planes separated by one period, *z* = 15 µm (upper) and *z* = 65 µm (lower). (e, f) Transverse polarization ellipse maps overlying on intensity distributions. (g, h) Corresponding Stokes skyrmion textures with calculated skyrmion numbers. (i, j) Stokes skyrmion textures of the vector fields. The central color bar applies to panels (g), (h), (i), and (j).

This framework allows us to tune the axial period Λ by engineering the pair of *NA*s, while ensuring multiple complete cycles of Stokes texture evolution are observable within the available non-diffracting region. To verify this theoretical model, we performed numerical simulations with parameters chosen for clear demonstration: NA_1_ = 0.4 and NA_2_ = 0.465, which yields a longitudinal period of Λ = 50 μm. A quantitative analysis of this periodic precession is presented in Figure 2(a–d), performed at the radial ring where *S*_3_ = 0. Figure 2(a) provides a direct visualization of the Stokes parameter equations, plotting the normalized *S*_1_ and *S*_2_ values as a function of *z*. The clear sinusoidal nature of the curves and their quadrature phase relationship are in perfect quantitative agreement with the theoretical cosine and sine dependence, confirming the uniform precession of the transverse Stokes vector. While Figure 2(c) illustrates this evolution over one full period, Figure 2(b) reveals a fundamental half-period symmetry in the relative dynamics where its singularities correspond to the zero-crossings of *S*_1_. The path is confined to the equatorial plane, a direct consequence of the energy balance between the two constituent beams at this specific radius, as dictated by the *S*_3_ = 0 condition, and traces a complete circle, providing a compelling visual analogy for the periodic precession.

Beyond the evolution at a single radial point, our model predicts that the entire two-dimensional spatial structure of the polarization field should be periodic. To provide direct visual evidence of this, we compare the field’s texture at two distinct axial planes separated by exactly one period: *z* = 15 μm and *z* = 65 μm. Figure 2(e) and (f) present the calculated polarization ellipse maps for these two planes. The near-perfect congruence between the two maps, where the orientation, ellipticity, and handedness of the polarization at every corresponding pixel are identical, provides the first layer of powerful visual evidence for this structural periodicity.

This periodicity is further confirmed by examining the topological texture of the field, visualized as a Stokes skyrmion in Figure 2(g) and (h). An optical skyrmion is a vector field with nontrivial topology, and its texture is quantified by a topological invariant:

$$N_{sk}=\frac{1}{4\pi }\int s\cdot \left(\frac{\partial s}{\partial x}\times \frac{\partial s}{\partial y}\right)dxdy$$

which is the skyrmion number of the vector field. The calculated values for the two planes, *N*_sk_ = 0.97 and *N*_sk_ = 0.99 respectively, confirm the presence of a stable, topologically non-trivial structure with integer skyrmion number (*N*_sk_ ≈ 1) that is preserved during propagation. Furthermore, the specific texture, whether it is Bloch-type (vectors spin tangentially) or Néel-type (vectors point radially), is determined by the initial phase between the two superposed beams, adding another degree of control. The striking similarity between the two skyrmion portraits provides robust confirmation that the entire topological configuration is periodically restored.

Figure 2(i) and (j) offer an intuitive three-dimensional rendering of the Stokes vector field at the two selected planes. This representation provides a clear picture of the vector stokes texture of the skyrmion. The visual identity of the two structures serves as the definitive confirmation that the entire complex vector field is fully restored after propagating one longitudinal period Λ. This comprehensive analysis, demonstrating rigorous agreement between analytical formulas, quantitative plots, and direct visualizations, provides a complete and self-consistent picture of the field’s deterministic and periodic longitudinal evolution. Such consistency across analytical and visual domains further underscores the robustness of the proposed mechanism in maintaining topological structure over extended propagation distances.

### 2.3 Metasurface design for generating and modulating skyrmions

Having clarified the underlying principles governing the longitudinal evolution of optical skyrmions, we now explore how to deliberately generate and manipulate such topological spin textures in practical settings. Metasurfaces provide a flexible and effective means of translating the intrinsic physics of skyrmions into controllable field structures. By carefully adjusting unit cell parameters across multiple dimensions and tailoring phase responses at the subwavelength scale, one can design devices that support multi-channel and multifunctional operations. Utilizing the orthogonality of LCP and RCP enables polarization multiplexing, where distinct spin states can be independently addressed.

Among various structured beams, Bessel beams are especially attractive due to their ability to maintain shape during propagation and to reconstruct after partial obstruction, making them suitable for a wide range of optical tasks. However, conventional metasurfaces that rely solely on propagation or geometric phase tend to generate Bessel beams with static wavefronts. To address this limitation, we incorporate multidimensional design strategies that allow independent control over spin states and topological charges, leading to tunable Bessel beam generation across polarization channels. This framework opens up possibilities for dynamically reconfigurable light fields in advanced photonic applications.

The underlying principle is derived from the Jones matrix and propagation phase [36]. We express the eigen-fields as

$$\begin{array}{c} E_{LCP}=\left[\begin{array}{c} E_{x}\\ E_{y} \end{array}\right]=\frac{1}{\sqrt{2}}\left[\begin{array}{c} 1\\ i \end{array}\right]\\ E_{RCP}=\left[\begin{array}{c} E_{x}\\ E_{y} \end{array}\right]=\frac{1}{\sqrt{2}}\left[\begin{array}{c} 1\\ -i \end{array}\right] \end{array}$$

Designing the nanostructure as a half-wave plate yields the Jones matrix

$$J_{\frac{\lambda }{2}}=\left[\begin{array}{cc} 1 & 0\\ 0 & -1 \end{array}\right]e^{i\varphi_{x}}$$

where *φ*_*x*_ is the propagation phase accumulated along the x-polarized eigen-direction.

Rotating the element by an angle *θ* gives the rotation matrix

$$R\left(\theta \right)=\left[\begin{array}{cc} \cos\theta & \sin\theta \\ -\sin\theta & \cos\theta \end{array}\right]$$

For an incident LCP field, the transmitted field becomes

$$E_{out}=J\left(\theta \right)E_{LCP}=\frac{1}{\sqrt{2}}\left[\begin{array}{c} e^{i2\theta }\\ -ie^{i2\theta } \end{array}\right]e^{i\varphi_{x}}=E_{RCP}e^{i\varphi_{x}}e^{i2\theta }$$

Conversely, for an incident RCP field,

$$E_{out}=J\left(\theta \right)E_{RCP}=\frac{1}{\sqrt{2}}\left[\begin{array}{c} e^{i-2\theta }\\ ie^{i-2\theta } \end{array}\right]e^{i\varphi_{x}}=E_{LCP}e^{i\varphi_{x}}e^{-i2\theta }$$

Hence, the geometric-phase principle yields

$$\begin{array}{c} \varphi_{LCP}=\varphi_{x}+2\theta \\ \varphi_{RCP}=\varphi_{x}-2\theta \end{array}$$

Here *φ*_*x*_ is the propagation phase for x-linearly polarized incidence, and ±2*θ* is the Pancharatnam–Berry phase imparted by rotation.

From a control-parameter perspective, independent phase modulation of the two circular polarizations requires two degrees of freedom-analogous to solving a system of two linear equations. Because *φ*_*x*_ and ±2*θ* are mutually independent, polarization-multiplexed metasurfaces are theoretically viable.

For Bessel beams, the required phase profile is

$$\varphi_{bessel}^{n}=-\frac{2\pi }{\lambda }\sqrt{x^{2}+y^{2}}NA+n\cdot \arctan\left(\frac{y}{x}\right)$$

where *n* denotes the Bessel order and NA is the numerical aperture.

**Figure 3:**
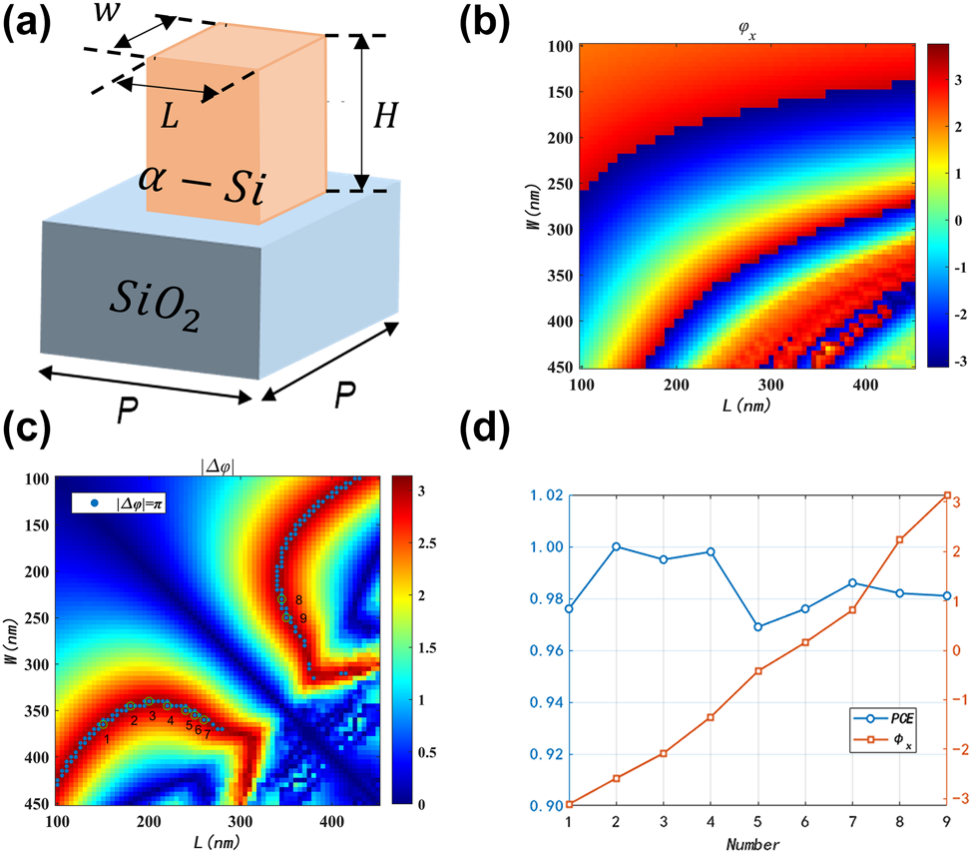
Metasurface nanofin design for optical skyrmions. (a) Three-dimensional schematic of α-Si (α-silicon) nanopillars on a SiO_2_ substrate, while the key geometric parameters are length *L*, width *W*, height *H*, and orientation angle *θ*. (b) Simulated transmission phase *φ*_*x*_ for *x*-linearly polarized light as functions of *L* and W (*H* fixed). (c) Phase difference Δ*φ* between the *x*- and *y*-polarized responses of the unit cell, and the highlighted points satisfy $\left|\Delta \varphi \right|=\pi$ . (d) Selected nanofins exhibit a full 2*π* phase coverage for *x*-polarization with near-unity polarization conversion efficiency (PCE). Simulations are performed at *λ* = 1550 nm.

Guided by the design principles set out above, the unit cell must furnish independent control of the propagation phase *φ*_*x*_ and the geometric phase (±2*θ*) so that left- and right-handed circular polarizations can be orthogonally multiplexed. We performed a systematic three-dimensional finite-difference time-domain (FDTD) sweep on silicon square pillars. All pillars were kept at a constant height *H* = 1200 nm, a value sufficient to span a full 0–2*π* propagation-phase excursion at the operating wavelength of 1550 nm while maintaining sub-wavelength cross-sections to suppress higher-order diffraction. The square side length *L* and width *W* were both varied from 100 nm to 450 nm in 5 nm increments. For each geometry we extracted the complex transmission coefficients *t*_*xx*_ and *t*_*γγ*_ under *x*- and *y*-linearly polarized incidence and derived:

The propagation phase *φ*_*x*_ is defined as the phase angle of *t*_*xx*_, while the phase anisotropy Δ*φ* is the absolute difference between the phase angles of *t*_*xx*_ and *t*_*γγ*_. A representative subset of geometries was further evaluated by Finite-Difference Time-Domain for the polarization-conversion efficiency (PCE) to quantify how closely each element approximates an ideal half-wave plate. The complete data set is presented in Figure 3. The upper panel plots *φ*_*x*_ versus *L* together with the corresponding transmittance $T=\left({\left|t_{xx}\right|}^{2}+{\left|t_{\gamma \gamma }\right|}^{2}\right)/2$ ; the lower panel overlays Δ*φ* (solid line) and PCE (dashed line). Regions where Δ*φ* = *π* rad and PCE > 0.97 are highlighted that simultaneously satisfy the half-wave-plate criterion and maximize throughput, providing the essential foundation for the subsequent circular-polarization-multiplexed Bessel-beam generator.

Our designed metasurface physically realizes the longitudinally evolving skyrmion field by simultaneously generating and coaxially superposing two distinct Bessel beams. Based on our theoretical design, we selected a 0th-order Bessel beam with NA_1_ = 0.4 for the RCP channel and a 1st-order Bessel beam with NA_2_ = 0.65 for the LCP channel, a combination engineered to produce a longitudinal evolution period of Λ = 10 μm. A dielectric metasurface, constructed from the library of optimized silicon nanopillars described previously, provides the ideal platform to achieve this complex, polarization-multiplexed functionality. By precisely arranging the pillars according to their individual side lengths and rotation angles, the metasurface collectively leverages both propagation and geometric phase to impart the two required, independent phase profiles. The performance of the final device was then validated through FDTD.

**Figure 4:**
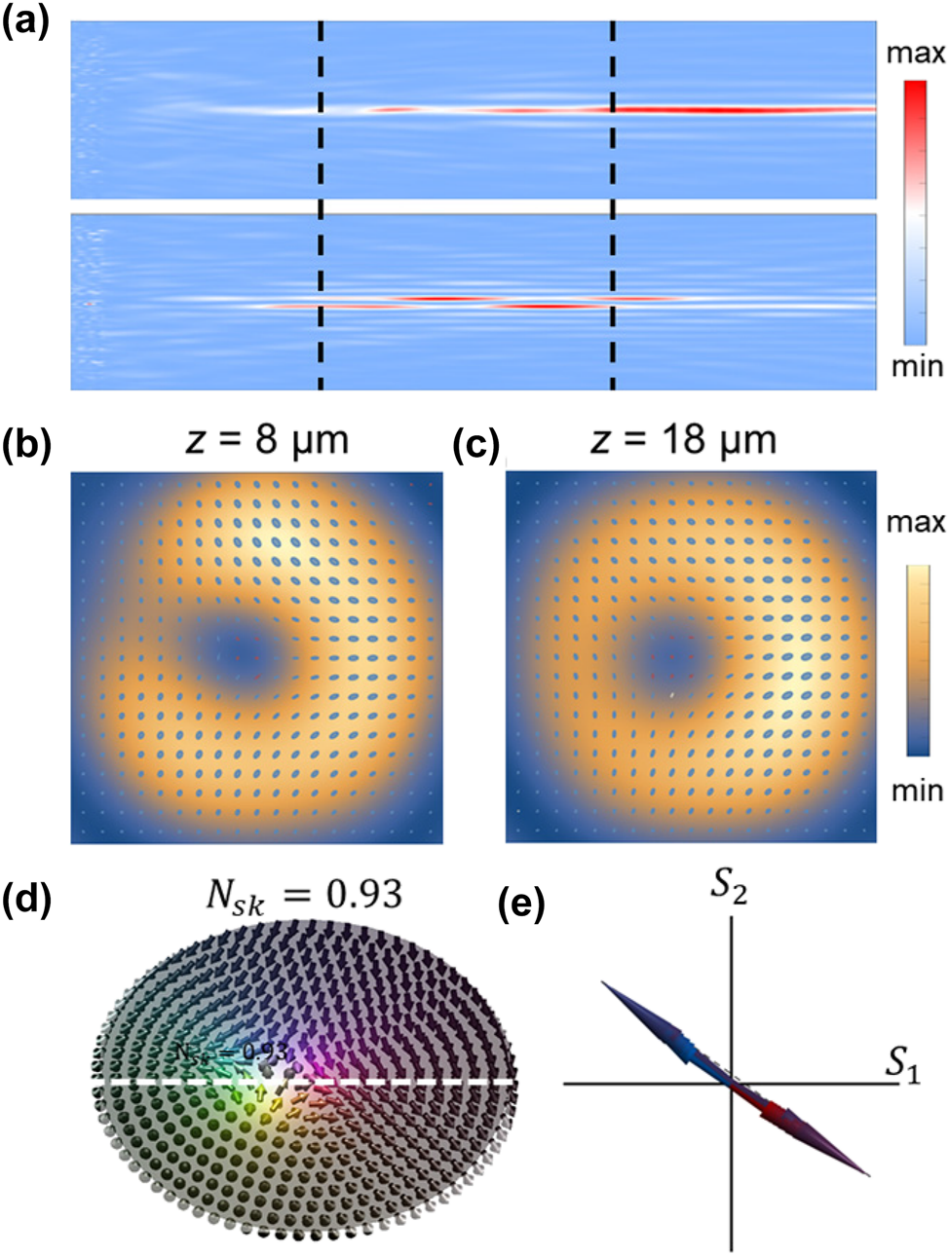
The metasurface-generated field and longitudinal evolved skyrmions. (a) Simulated intensity distributions in the *xz* profile for the 0th-order (top), 1st-order (bottom). (b, c) Transverse polarization ellipse maps overlying on intensity distributions of the superimposed fields at planes in two periods. (d) Three-dimensional visualization of the Stokes skyrmion texture. (e) Transverse Stokes vectors (*S*_1_, *S*_2_) extracted along the horizontal axis.

The simulation results, presented in Figure 4, confirm that the designed metasurface generates the desired longitudinally evolving Stokes skyrmion field. Figure 4(a) shows the intensity distribution of the generated RCP (top panel) and LCP (bottom panel) components in the *xz* plane. Both channels clearly exhibit the characteristic non-diffracting behavior of Bessel beams, validating the independent phase control. The superposition of these two beams creates a total field whose polarization texture evolves with the designed period of Λ = 10 μm.

To demonstrate this longitudinal periodicity, we examined the transverse polarization profiles at two axial planes separated by exactly one period: *z* = 8 μm and *z* = 18 μm. The resulting polarization ellipse maps are shown in Figure 4(b) and (c), respectively. As predicted, the two distributions are qualitatively identical, showing that the entire spatial polarization structure of the field is precisely restored after propagating a distance Λ. This periodic restoration means both planes share a common topological structure, which is visualized as a three-dimensional Stokes skyrmion in Figure 4(d). The calculated skyrmion number, *N*_sk_ = 0.93, confirms the generation of a stable, topologically non-trivial field.

The field generated by the metasurface faithfully reproduces the intrinsic structural properties derived from our analytical model. As predicted by the term Δ*l* *ϕ* in our Stokes parameter equations, the orientation of the transverse Stokes vector is a function of the azimuthal angle *ϕ* but is independent of the radial coordinate. Figure 4(e) confirms this property by showing that the vectors with the same *ϕ* = 0 or *ϕ* = *π* extracted along the horizontal line share an identical orientation, varying only in magnitude. This result demonstrates that our metasurface design successfully translates the abstract principles of skyrmion formation into a physically realizable optical field. In summary, the simulation results provide compelling evidence that by leveraging a multi-dimensional design strategy, a single, static metasurface can generate complex, multiplexed vector fields and precisely control their periodic evolution of the skyrmionic structure in the longitudinal dimension.

## 3 A method for high-precision axial positioning

Our proposed method for high-precision axial positioning is founded upon a “coarse-and-fine” measurement strategy, meticulously designed to overcome the inherent ambiguity of periodic signals and thereby achieve absolute position determination over an extended axial range. The motivation for this approach arises from a central challenge in modern optical metrology: the concurrent demand for both high precision and a large, unambiguous operational range. While established techniques such as confocal microscopy offer excellent axial sectioning, their effective range is fundamentally limited by the objective’s depth of field. Conversely, methods like white-light interferometry provide exceptional sub-nanometer precision but are often restricted to reflective surfaces, require mechanical scanning, and can be highly susceptible to environmental vibrations. Our method circumvents these limitations by developing a single-shot, non-contact technique based on the propagation dynamics of a specially engineered structured light field. By encoding two complementary axial features – one monotonic and one periodic – our method resolves the sensitivity-versus-range trade-off and establishes a scalable metrology framework.

The foundation of the scheme is the introduction of a fiducial reference plane, z_*s*_, which serves as a global origin for all measurements. This plane is physically defined as the first axial location where the transverse Stokes vector is precisely aligned with the *S*_1_ axis (i.e., *S*_1_ = 1, *S*_2_ = 0). Referencing this plane allows for a flexible operational range that is not strictly tied to the beam waist. The absolute axial position *Z* is then calculated as $Z=z_{s}+\left(K+\delta \right)\cdot \Lambda$ , where *K* is an integer coarse index identifying the specific longitudinal period, *δ* is a fine intra-period fraction, and Λ is the engineered longitudinal period. By assigning distinct values, NA_1_ = 0.4 and NA_2_ = 0.65, to the two beams, their longitudinal wavenumbers differ by Δ*k*_*z*_ giving rise to a controlled beating effect. The resulting longitudinal period is $\Lambda =\frac{2\pi }{\left|k_{z1}-k_{z2}\right|}=10\;\mu \text{m}$ which governs the cyclic evolution of the field’s polarization. In our implementation, the fiducial plane is located at *z*_*s*_ = 7.8 μm, and the operational period is Λ = 10 μm. This dual-coordinate representation is conceptually analogous to a hybrid digital–analog encoding scheme. The integer *K* provides discrete, robust localization across multiple periods, akin to reading the main integer millimeter scale on a vernier caliper. Concurrently, the continuous variable *δ* offers fine positioning with subwavelength resolution, analogous to the caliper’s sliding vernier scale that interpolates between the main markings. This hybrid approach ensures that the system’s global accuracy is not compromised by the periodic nature of its high-sensitivity component.

This engineered superposition yields two key axial features. The first is a complex polarization texture, which in each transverse plane forms an optical skyrmion, a topologically non-trivial, particle-like vector field. As the beam propagates, this skyrmionic texture undergoes a continuous, periodic transformation, cyclically evolving between Bloch-type and Néel-type configurations with the period Λ. The second feature is a monotonic on-axis center intensity. This intensity profile is leveraged through a straightforward, one-time calibration process. In practice, the metasurface plane is focused and defined as the reference position *z* = 0, and an initial offset *z*_*s*_ is introduced to set the start of the operational range. A set of calibration points are then sampled to precisely map the monotonic intensity curve, partitioning the entire range into unambiguous periods by establishing clear intensity thresholds at their boundaries. Once this intensity map is established, any subsequent measurement becomes absolute. The combination of the globally unambiguous but low-resolution intensity feature and the highly sensitive but cyclically ambiguous polarization feature is the cornerstone of our method’s high performance over the operational range, which for this design covers two consecutive periods from 7.8 µm to 27.8 µm. Furthermore, the calibration process enables a powerful in-situ correction of the evolution period Λ. This addresses potential discrepancies between the designed value and the actual period arising from fabrication tolerances or experimental conditions. By measuring the polarization states at two precisely known axial positions, the resulting angular rotation of the Stokes vector provides the necessary data to calculate the actual period with high accuracy. This corrected period is then used for all subsequent measurements, ensuring the system’s reliability and precision in practical applications.

**Figure 5:**
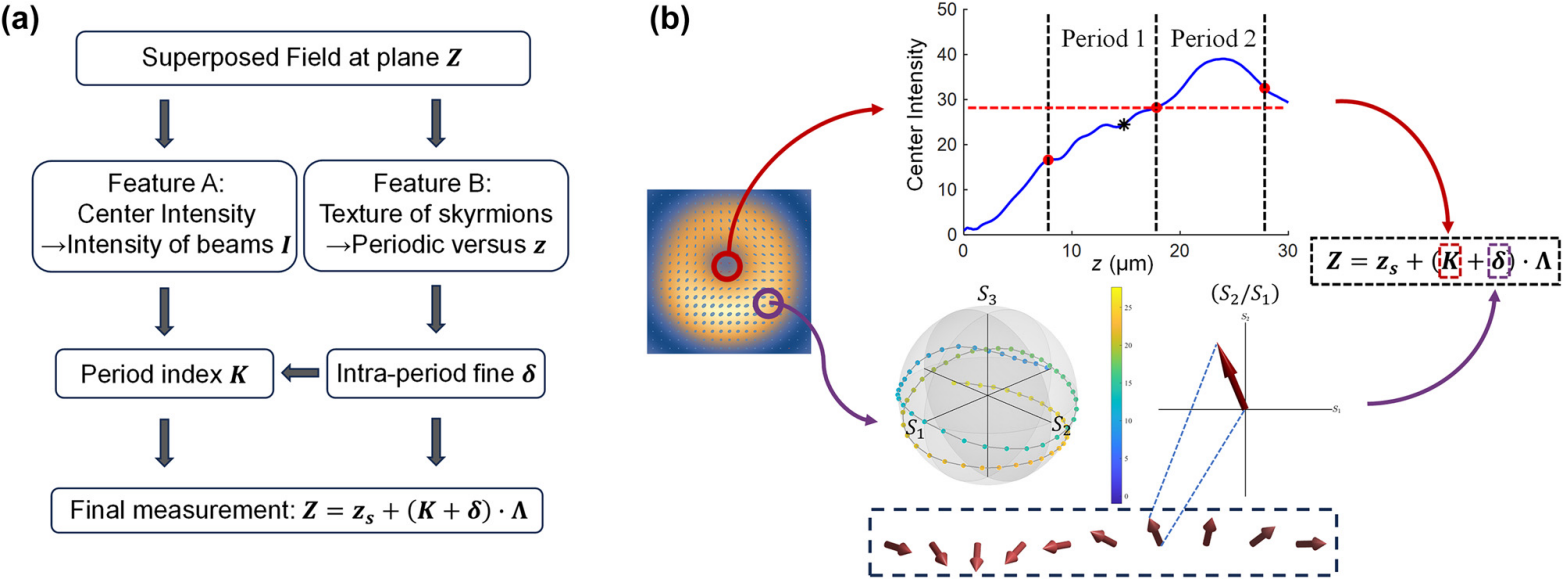
Principle and workflow of the coarse-and-fine axial positioning method. (a) Workflow of the dual-feature method, where the monotonic center intensity determines the coarse period index *K*, and the periodic skyrmion texture determines the fine intra-period fraction *δ*. The fine fraction *δ* provides an additional correction to *K*, improving system robustness. (b) Example of the demodulation process. The measured on-axis intensity (black star) falls below a pre-calibrated inter-period threshold, identifying the period index. The corresponding polarization state (visualized as a vector and on the Poincaré sphere) is mapped to the fine fraction *δ*. Both are combined to calculate the absolute position *Z*.

The demodulation process to extract *z* from a captured field profile is executed as a two-step algorithm. First, the coarse index *K* is determined. This is achieved by isolating and measuring the on-axis intensity. Specifically, this refers to the total intensity of the superimposed field, integrated over a small, pre-defined area centered on the system’s optical axis (r = 0). As shown in the top panel of Figure 5(b), the intensity at the boundary between Period 1 and Period 2 serves as a single, critical threshold. For an unknown measurement, if its on-axis intensity is lower than this threshold, it is unambiguously assigned to the first period (*K* = 0). Conversely, if the intensity is higher, it is assigned to the second period (*K* = 1). The example in the figure shows a test point (in blue) whose intensity falls below the threshold, correctly identifying it as being in Period 1. Second, the intra-period coordinate *δ* is extracted from the polarization state. This requires a full Stokes polarimetry measurement of the captured field to determine the spatially resolved parameters *S*_1_ and *S*_2_. The transverse Stokes vector (*S*_1_, *S*_2_) rotates by a full 2*π* within each period Λ, with its orientation angle $\theta =\text{atan}(S_{2}/S_{1})=\Delta k_{z}z$ , directly corresponding to the orientation of the local linear polarization ellipse. The intra-period fraction *δ* is then given by the robust four-quadrant arctangent mapping, $\delta =\mathrm{mod}(atan2(-S_{2},S_{1})/2\pi )$ , which ensures a continuous, one-to-one mapping of the polarization angle onto the fractional range $\left[0,1\right)$ , providing high-resolution positional information.

An essential feature of our method is its inherent robustness, which arises from the ability to cross-validate the intensity and polarization measurements. Small oscillations in the intensity profile, due to finite aperture effects or beam imperfections, could otherwise introduce ambiguity near period boundaries. For example, an intensity value *I*_z_ might be consistent with both a position at the end of Period 1 (e.g., *z* = *z*_*s*_ + 0.9Λ) and one at the beginning of Period 2 (e.g., *z* = *z*_*s*_ + 1.1Λ). The polarization measurement, however, definitively resolves this: the former case would yield *δ* ≈ 0.9, while the latter would yield *δ* ≈ 0.1, allowing the correct period index *K* to be confirmed. This self-correcting mechanism significantly enhances tolerance to experimental noise and systematic errors, such as those arising from detector noise or minor optical aberrations. The ultimate precision of our method is determined by the ability to resolve changes in the polarization state. The axial position uncertainty, Δ*z*, is given by $\Delta z=\frac{\Lambda }{2\pi }\Delta S/\sqrt{S_{1}^{2}+S_{2}^{2}}$ , where Δ*S* is the sensitivity of the Stokes polarimeter. This expression reveals that the system’s precision is directly tunable via the engineered period Λ. With state-of-the-art polarimetry instruments, this allows for a theoretical precision in the sub-nanometer range, while a nanometer-scale precision can be achievable in practical single-shot imaging configurations, based on full Stokes polarization cameras [41].

Beyond demonstrating an abstract principle, this framework offers a broadly applicable and scalable metrology tool. The measurement range can be extended simply by adding more calibration points to the monotonic intensity curve, while the precision, determined by the period Λ, can be tuned by adjusting the NAs of the initial beams. Since the method relies solely on intensity and polarization detection, it is compatible with existing microscopy setups. Unlike scanning-based methods, our approach enables single-shot axial localization. Furthermore, the common-path nature of the superimposed beams provides enhanced stability against environmental vibrations compared to traditional interferometric techniques. In practice, the scheme enables precise, absolute, and non-contact axial localization with potential applications.

## 4 Discussion

This work presents a practical method to control the longitudinal evolution of optical skyrmions using a single dielectric metasurface. The method can be further improved by simply modifying the metasurface design or using inverse design strategies to enhance its functionality [42], [43], [44], [45], [46]. For example, increasing the NA of the beams allows for tighter focusing, which generates smaller skyrmion spots, improves lateral resolution in sensing, and supports higher density in data storage and transmission. Adjusting the size of the input Gaussian beam, along with enlarging the metasurface area, can help extend the stable propagation distance of the skyrmions, making it possible to achieve more evolution cycles. By tuning the relative propagation parameters of the two beams, the polarization evolution period can be adjusted, providing a topological light source with high precision and long range for applications such as precision metrology [47]. The proposed device is fully compatible with conventional microfabrication processes used in integrated photonic platforms [48]. The flat and compact structure of the metasurface also makes it suitable for dynamic control. By introducing tunable materials such as phase-change media [49], [50] or liquid crystals, the NA can be actively adjusted in real time, enabling flexible control over the evolution characteristics of skyrmions. This is particularly useful in practical scenarios where different resolutions or beam behaviors are required at different moments.

We have successfully proposed and numerically validated a novel method that enables the precise, periodic modulation of a skyrmion’s Stokes properties along the longitudinal axis. The core mechanism relies on the coaxial superposition of a zero-order LCP beam and a first-order RCP beam, critically engineered with distinct NAs by a single, multi-functional metasurface. This difference in NAs creates a well-defined wavevector mismatch, which governs the periodic beating of the polarization state. The evolution period is therefore a designable parameter, offering full control over the field’s longitudinal behavior. To showcase the practical utility of this principle, we designed a high-precision displacement sensing method. This scheme uniquely combines a monotonic intensity signal for coarse, unambiguous period indexing with the highly sensitive periodic polarization evolution for fine, sub-wavelength measurements, enabling absolute position determination from a single snapshot and overcoming the limitations of traditional scanning techniques. Our method provides a versatile platform for precision metrology and optical information technologies. By deterministically controlling the longitudinal evolution and polarization of optical skyrmions, it enables applications in high-resolution displacement sensing, polarization-multiplexed communication, and structured-light data encoding, offering a compact route toward integrable photonic devices.
